# Metastasising Fibroblasts Show an HDAC6-Dependent Increase in Migration Speed and Loss of Directionality Linked to Major Changes in the Vimentin Interactome

**DOI:** 10.3390/ijms23041961

**Published:** 2022-02-10

**Authors:** Caroline A. Evans, Hyejeong Rosemary Kim, Sarah C. Macfarlane, Poppy I. A. Nowicki, Carsten Baltes, Lei Xu, Jerker Widengren, Franziska Lautenschläger, Bernard M. Corfe, Annica K. B. Gad

**Affiliations:** 1 Department of Chemical and Biological Engineering, University of Sheffield, Mappin St, Sheffield S1 3JD, UK; caroline.evans@sheffield.ac.uk; 2 Department of Oncology and Metabolism, The Medical School, University of Sheffield, Beech Hill Road, Sheffield S10 2RX, UK; h.r.kim@sheffield.ac.uk (H.R.K.); scmacfarlane1@sheffield.ac.uk (S.C.M.); pianowicki1@sheffield.ac.uk (P.I.A.N.); 3 Experimental Physics, NT Faculty, D2 2, Saarland University, 66123 Saarbrücken, Germany; carstenbaltes@gmx.de (C.B.); f.lautenschlaeger@physik.uni-saarland.de (F.L.); 4 Department of Applied Physics/Experimental Biomolecular Physics, KTH Royal Institute of Technology, SE-100 44 Stockholm, Sweden; lxu@kth.se (L.X.); jwideng@kth.se (J.W.); 5 Population Health Sciences Institute, Human Nutrition Research Centre, Faculty of Medical Sciences, Newcastle University, Newcastle NE2 4HH, UK; Bernard.Corfe@newcastle.ac.uk; 6 Madeira Chemistry Research Centre, University of Madeira, 9020105 Funchal, Portugal

**Keywords:** oncogenes, metastasis, cell migration, histone deacetylase 6, vimentin, vimentin interactome, fibroblasts, genome-wide mass spectrometry, stimulated emission depletion microscopy

## Abstract

Metastasising cells express the intermediate filament protein vimentin, which is used to diagnose invasive tumours in the clinic. We aimed to clarify how vimentin regulates the motility of metastasising fibroblasts. STED super-resolution microscopy, live-cell imaging and quantitative proteomics revealed that oncogene-expressing and metastasising fibroblasts show a less-elongated cell shape, reduced cell spreading, increased cell migration speed, reduced directionality, and stronger coupling between these migration parameters compared to normal control cells. In total, we identified and compared 555 proteins in the vimentin interactome. In metastasising cells, the levels of keratin 18 and Rab5C were increased, while those of actin and collagen were decreased. Inhibition of HDAC6 reversed the shape, spreading and migration phenotypes of metastasising cells back to normal. Inhibition of HDAC6 also decreased the levels of talin 1, tropomyosin, Rab GDI β, collagen and emilin 1 in the vimentin interactome, and partially reversed the nanoscale vimentin organisation in oncogene-expressing cells. These findings describe the changes in the vimentin interactome and nanoscale distribution that accompany the defective cell shape, spreading and migration of metastasising cells. These results support the hypothesis that oncogenes can act through HDAC6 to regulate the vimentin binding of the cytoskeletal and cell–extracellular matrix adhesion components that contribute to the defective motility of metastasising cells.

## 1. Introduction

Metastasising tumour cells show increased levels of the intermediate filament protein vimentin, which has, therefore, been used to diagnose invasive tumours in the clinic for decades. Vimentin is a canonical marker of epithelial-to-mesenchymal transition (EMT), a process that occurs during embryogenesis, wound healing and cancer in which stationary cells change from a round to an elongated cell shape and become migratory. Recent findings have shown that vimentin is not only a passive marker of carcinomas and EMT, but might also induce changes in cell shape, adhesion and migration—as well as promoting the invasion of tumour cells [1,2,3,4,5,6]. In particular, vimentin expression in epithelial cells is required and sufficient for the change from a round to an elongated cell shape, and vimentin is required for directional cell migration [7,8]. Vimentin is also required for the mechanical and adhesion properties of cells, and the metastatic spread of lung cancers [1,7,8,9]. This might be due, in part, to the promotion of single cell migration by vimentin, which regulates the functions of the actin microfilament and microtubule filamentous systems [9,10].

However, the molecular mechanisms through which vimentin regulates cell shape and motility remain incompletely understood. In addition to the regulation of the actin microfilament and microtubule systems, vimentin is also likely to control cell–matrix adhesions. An increasing number of observations have suggested that vimentin regulates both the inside-out control of the growth of initial focal complexes and the outside-in signalling required to form mature and large focal adhesions. For example, long and mature filaments localise to the base of relatively large cell–matrix adhesions, and this localisation to focal adhesions stabilises and promotes the mechanical strength of these adhesions. The transient depolymerisation of filamentous vimentin occurs locally, just prior to the formation of lamellipodia [11]. Accordingly, in a recent study by Ostrowska-Podhorodecka et al., binding of the cell-adhesion component talin 1 to vimentin filaments shifted the inside-out to outside-in signalling at cell–matrix adhesions, which resulted in focal adhesion maturation [12]. The loss of vimentin reduced the number and size of cell–matrix adhesions, as well as the levels of the cell-adhesion molecule integrin β1 and the F-actin cross-linker filamin A [13]. Together with the observation that polymerisation-deficient vimentin suppresses the growth of cell–matrix adhesions via a mechanism that requires calcium-dependent, non-lysosomal calpain proteases [14], this is in line with a previously proposed model that suggests that competition between the calpain-mediated proteolysis of talin 1 and filamin A at focal adhesions is required for the growth of focal adhesions [15]. Vimentin can also promote the production of the extracellular matrix [16,17], which suggests an indirect vimentin-dependent regulation of outside-in signalling. However, the mechanisms by which vimentin regulates and integrates cell–matrix adhesion, cell shape, and motility in health and disease remain to a large extent unclear.

Vimentin is the main intermediate filament system in fibroblasts. We have previously shown that in fibroblasts, oncogenes reduce the size and increase the numbers of micrometer-sized cell–matrix adhesions [18]. This results in a more homogenous distribution of nanoscale cell-matrix adhesions, and these changes are linked to a more entangled network of vimentin filaments. The focus of this work is to describe how HDAC6 influences the spatial organization of vimentin, and the downstream consequences for intracellular signalling and cell migration—but not on the protein levels of vimentin. HDAC6 is a protein required for the RasV12 oncogene to oncogenically transform cells, and for the formation of aggresomes, cytokinesis and EMT; it is currently a key target for the development of drugs against cancer [19]. The change in cell shape from circular to elongated that accompanies EMT caused by increased levels of vimentin is also observed upon the inhibition of HDAC6 [20]. In many cell types, the directionality of cell migration correlates with migration speed [21]. We hypothesised that in our fibroblast system, oncogenes act via HDAC6 to change the vimentin interactome, and thereby the speed and the directionality of cell migration—as well as the coupling between cell speed and directionality.

These data suggest that in our fibroblast system, the transformation of Bj primary fibroblasts to metastasising cells is accompanied by the loss of the elongated cell shape, reduced cell spreading, increased cell migration speed, reduced directionality, stronger correlation between cell migration speed and reduced directionality, and changes in the nanoscale spatial distribution and the protein interactome of vimentin via an HDAC6-dependent mechanism. These observations are in line with the hypothesis that oncogenes promote the dysregulation of cell motility and metastasis via HDAC6-mediated regulation of the spatial distribution and binding of vimentin to components of cell-matrix adhesions.

## 2. Results

### 2.1. SV40T and H-RasV12 Change the Spatial Organisation of Vimentin More Than That of Actin and Tubulin

For cells to migrate from a tumour to a different site, the cell matrix adhesion and motility of the cells need to change. Although the cytoskeleton is the main determinant of these cell properties, how it contributes to metastasis is not fully understood. To determine how the three filamentous systems change during the transformation from primary fibroblasts to metastasising cells, we analysed the spatial distributions of actin microfilaments, microtubules and vimentin intermediate filaments in an isogenically matched stepwise cell model of four human cell variants that represent the transformation from primary fibroblasts to metastasising cells. Briefly (see also Materials and Methods), these are neonatal Bj human dermal fibroblasts, transformed using three well-defined genetic elements: the telomerase catalytic subunit (hTERT) in combination with two oncogenes (simian virus 40 large-T oncoprotein; V12 oncogenic allele of H-ras) [22]. These variants were as follows: (i) primary Bj fibroblasts; (ii) immortalised Bjhtert fibroblasts; (iii) cell cycle-defective BjhtertSV40T fibroblasts; and (iv) BjhtertSV40TRasV12 fibroblasts; the last of these form tumours, invade into the surrounding environment (Supplementary Figure S1), and metastasise in immunodeficient mice [23,24].

**Figure 1:**
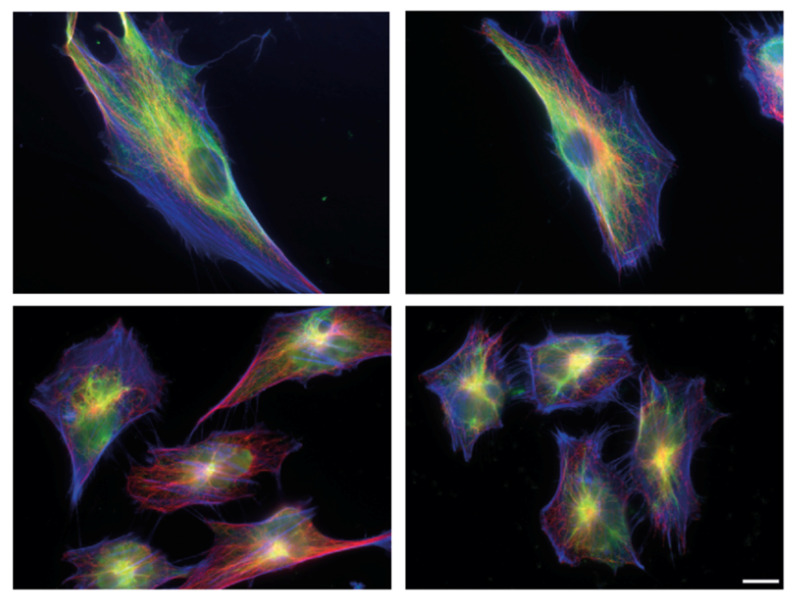
Cytoskeletal organisation and shape of human fibroblasts for the different stages of metastatic transformation. Immunoflourescence staining followed by Epiflourescence 2D microscopy showing Bj primary fibroblast (**top left**), Bjhtert cells (**top right**), BjhtertSV40T cells (**bottom left**) and metastasising BjhtertSV40TRasV12 cells (**bottom right**), showing vimentin (green), tubulin (red) and F-actin (blue). Scale bar, 10 μm.

Here, we observed that at each step, all three of the cytoskeletal systems changed (Figure 1, Supplementary Figure S2). In line with previously published transcriptome data, the most pronounced change was between cell types two and three: the immortalised cell variant; and the cell cycle-defective SV40T-expressing cell variant [25]. The SV40T-expressing variant showed actin microfilament-rich lamellipodia around the cell periphery, where the microtubules were more localised towards the cell centre. In line with the literature that microtubules guide the organisation of vimentin filaments, and that vimentin can promote the assembly of microtubules, the vimentin organisation followed the distribution of the microtubules [26,27]. While thin vimentin filaments were distributed throughout the cytoplasm in an even manner in the primary and immortalised cell variants, the vimentin in the oncogene-expressing and metastasising cells showed a striking redistribution towards the cell centre—a change that was even more pronounced than the changes in actin and microtubules. The phenotypes were particularly pronounced in the fourth variant, the RasV12-expressing metastasising cells (Figure 1; Supplementary Figure S2). These cytoskeletal changes were accompanied by significant changes in cell shape and size. The oncogene-expressing and metastasising cell types showed significantly reduced cell elongation and cell size, as compared to the control (primary) cells (Figure 1). Taken together, these data suggest that changes in the spatial organisation of vimentin have a role in oncogenic cell transformation and metastasis.

### 2.2. HDAC6 Is Required for the Shape and Motility of Metastasising Cells

**Figure 2:**
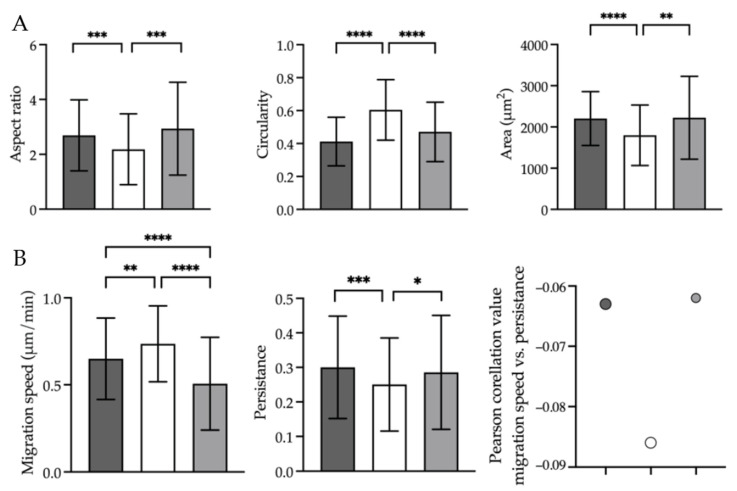
Oncogenes change cell shape and spreading area, speed and directionality of cell migration and speed–directionality coupling via HDAC6. (**A**) Epifluorescence 2D microscopy showing the aspect ratio (left), circularity (middle) and spreading area (right) of immortalised Bjhtert cells (dark grey) and metastasising BjhtertSV40TRasV12 cells without (white) and with (light grey) tubacin treatment. (**B**) Epifluorescence 2D live cell imaging showing the cell migration speed (left), cell migration persistence (middle) and Pearson’s correlation between migration speed and persistence (right), of cells as in (**A**). Data are means ± standard deviation of the total number of trajectories, shown in brackets, of Bjhtert (345), BjhtertSv40TRas without (149) and with tubacin (256), and three independent experiments. *, *p* ≤ 0.05; **, *p* ≤ 0.01; ***, *p* ≤ 0.001; ****, *p* ≤ 0.0001 (Mann–Whitney tests).

We next aimed to define the changes in cell shape, size and migration that accompany the metastatic process, and the role of HDAC6 in this process. To this end, we analysed cell shape and spreading area, the speed and directionality of cell migration, and the coupling between migration speed and directionality of metastasising cells without and with treatment with the highly potent and selective, reversible, cell-permeable HDAC6 inhibitor tubacin—as described in the Materials and Methods section. As compared to control fibroblasts, the metastasising cells showed a significantly reduced aspect ratio and increased circularity, which indicated the loss of the elongated fibroblast cell shape. This was accompanied by a reduction in the spreading area of the metastasising cells (Figure 2A), and the increased speed and reduced directionality of their migration. In particular, the correlation between increased speed and reduced directionality of migration was strengthened in the metastasising cells (Figure 2B; Supplementary Films S1 and S2). Here, tubacin-induced loss of HDAC6 activity in the metastasising cells reverted the changes in cell shape and spreading area to the controls. This also reduced the cell migration speed, increased the directionality of migration, and reduced the coupling between increased speed and reduced directionality of the metastasising cells to that of the control cells (Figure 2; Supplementary Film S3).

### 2.3. Inhibition of HDAC6 Activity Results in Loss of Nanoscale, Non-Filamentous, Short Versions of Vimentin at the Cell Periphery, and Formation of Cage-like Clusters/Asters of Filamentous Vimentin

**Figure 3:**
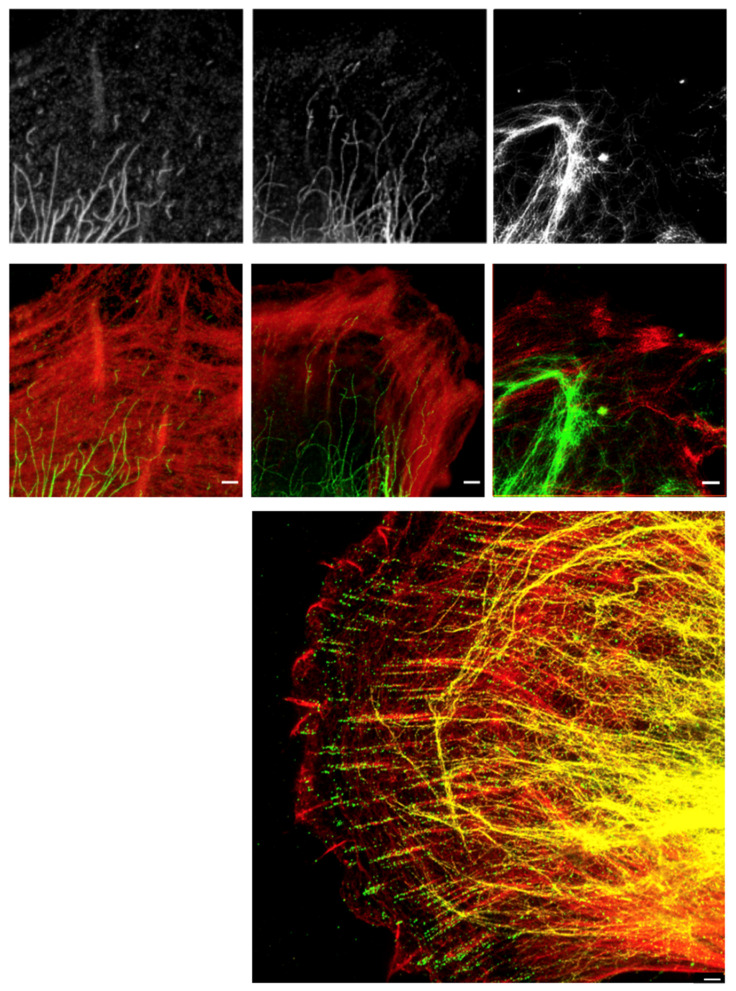
HDAC6 regulates the nanoscale spatial distribution of vimentin in oncogene-expressing and metastasising cells. Representative stimulated emission depletion (STED) super-resolution microscopy images of (**top**) immunofluorescence-stained Bjhtert cells (**left**; immortalised) and BjhtertSV40T cells (oncogene-expression) without (**middle**) and with (**right**) tubacin treatment, showing vimentin (white), (**middle panel**) vimentin (green) and F-actin (red) and (**lower panel**) a three-colour STED image of a BjhtertSV40TRasV12 cell showing EGFP-vimentin (yellow), phosphotyrosine (green) and F-actin (red). Scale bars: 1 μm.

To determine whether HDAC6 has a role in the regulation of the shape, spreading area and motility of cells via control of the spatial organisation of vimentin, we asked whether inhibition of HDAC6 activity changed the nanoscale spatial distribution of vimentin in oncogene-expressing cells. Upon inhibition of HDAC6 with tubacin (10 μM tubacin, 4 h, 37 °C), the nanoscale, non-filamentous vimentin units at the periphery of the oncogene-expressing cells were lost and were replaced by vimentin in a dense filamentous network, which often included nanoscale ‘knots’ or ‘asters’ at the cell periphery (Figure 3; Supplementary Figure S3). At the very periphery of the oncogene-expressing metastasising cells, there were non-filamentous units of vimentin, while (as reported previously [11,18]) filamentous vimentin was visible at the end of focal adhesions—oriented towards the cell centre—and not inside focal adhesions. In the area between focal adhesions and the cell nucleus, vimentin was localised in filaments, which were occasionally arranged in similar small vimentin ring-shaped clusters or asters, as observed upon inhibition of HDAC6 (Figure 3).

### 2.4. H-RasV12 and HDAC6 Activities Change the Binding of Cytoskeletal, Cell–Extracellular Matrix Adhesion Components to Vimentin Intermediate Filaments

**Figure 4:**
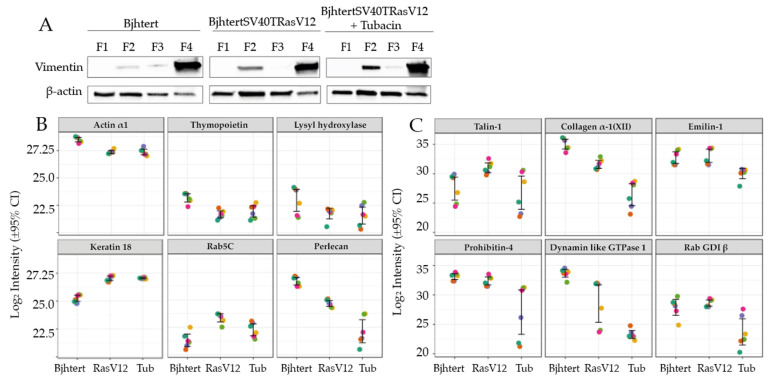
Vimentin interactome changes during metastatic transformation and upon inhibition of HDAC6 activity. Immortalised Bjhtert cells and metastasising BjhtertSV40TRasV12 cells without and with tubacin treatment. (**A**) Western blots for vimentin and actin in the cell fractions for the intermediate filament enrichment. (**B**,**C**) Mass spectrometry quantification of protein levels in the vimentin interactome in immortalised Bjhtert control cells and metastasising BjhtertSV40TRasV12 cells without (RasV12) and with (Tub) tubacin treatment. The six replicate measurements (two biological replicates, analysed in triplicate) are indicated by the coloured dots for each sample.

Vimentin has been proposed to act as a platform for the binding of proteins, in order to thereby regulate intracellular signalling [28,29]. To determine how oncogenes and HDAC6 affect the vimentin interactome, and to indicate any potential regulators of the vimentin-dependent control of cell shape and motility, we analysed the vimentin interactome in the metastasising BjhtertSV40TRasV12 cells without and with tubacin treatment, and in the immortalised Bjhtert cells as a control. The intermediate filament fraction of the cell variants was first enriched to yield four subcellular fractions: membrane bound/soluble protein; cytoskeletal; highly salt-soluble supernatant; and insoluble cytoskeleton (see Materials and Methods). The levels and quality of these four fractions are shown in Figure 4A, and the final fraction—fraction 4—was enriched in vimentin relative to β-actin (Figure 4A; Supplementary Figure S4). We then analysed the protein content of this vimentin-enriched fraction 4 using a label-free quantitative proteomics approach (Supplementary Dataset 1, with 555 proteins identified and relatively quantified by ≥2 unique peptides). In terms of overall composition, fraction 4 contained intermediate filament proteins, of which vimentin was the most abundant. Keratin 18, lamins A and B1, plectin and pinin were also detected. Proteins associated with integrin binding and interactions with the extracellular matrix—such as integrin β1, collagen α-1(VI), α-2(VI) and α-3(VI) chains, fibronectin, heparan sulphate proteoglycan core protein, talin 1 and CD44 antigen—were present. Bioinformatic comparisons of these data with online vimentin-interaction data (BIOGRID interaction database; https://thebiogrid.org/; accessed 20 October 2021) indicated that 50 of the proteins in the dataset are known interaction partners for vimentin, including plectin, keratin 18, the sequestosome-1 aggresome protein and the 14-3-3-ζ adaptor protein YWHAZ, which induces Rac1 activity and lamellipodia formation [30,31]. Together with the co-clustering of two biological and three technical replicates for each sample in the heatmap analysis (Supplementary Figure S5), these observations provide confidence in the dataset.

The vimentin interactome in the metastasising BjhtertSV40TRasV12 cells was thus characterised and compared to that of the immortalised Bjhtert cells (see Materials and Methods). We observed increased levels of 24 proteins and decreased levels of 23 proteins in the metastasising cells (Table 1, Supplementary Figures S5 and S6). BjhtertSV40TRasV12 cells showed increased levels of the intermediate filament protein keratin 18 and the small Rho GTPase Rab5C (Figure 4B), which is a protein required for the formation of focal adhesions and directed cell migration [32]. Furthermore, the vimentin interactome of these metastasising cells showed increased levels of 10 proteins associated with RNA metabolism (Supplementary Table S1), and of the glycolytic enzyme triosephosphate isomerase, the nuclear pore complex protein Nup153, and elongation factor 1-β. In contrast, the levels of the cytoskeletal components α-actin and thymopoietin were decreased (Figure 4B). The bioinformatic data mining of the interaction database identified α-actin and thymopoietin as known vimentin interactors. 

Proteins associated with the organisation of the extracellular matrix were also down-regulated in the vimentin interactome of the metastasising cells: collagen type XII; collagen processing protein procollagen-lysine,2-oxoglutarate 5-dioxygenase 1/lysyl hydroxylase; fibulin 2; and heparan sulphate proteoglycan 2 (Figure 4B). We also observed decreased levels of mitochondrial voltage-dependent anion channels 1,2, ubiquitin-processing protein RuvB-like 1, and proteasome 26S non-ATPase 6 subunit (Table 1). Taken together, in the vimentin interactome of the metastasising cells there were decreased levels of extracellular-matrix, cell-surface, cell-matrix adhesion, mitochondrial ion transport and ubiquitin-processing proteins, and increased levels of keratin 18, Rab5C and proteins involved in RNA metabolism. These data for selected proteins are shown in Figure 4B.

We then investigated whether the reversion of the cell shape, size and motility of metastasising cells to the control phenotype upon inhibition of HDAC6 was accompanied by a reversion of the vimentin interactome to that of the control cells. Tubacin treatment of BjhtertSV40TRasV12 cells resulted in increased levels of 9 proteins and decreased levels of 44 proteins. The global interactome in the metastasising cells was not reversed to that of the control cells; rather, the changes observed in the metastasising cells, as compared to control cells, were further enhanced by the tubacin treatment (Figure 2B, Supplementary Figures S5 and S6). Similar to oncogene expression, the tubacin treatment resulted in major decreases in the levels of cytoskeletal, cell-matrix adhesion, and extracellular matrix proteins in the vimentin interactome. However, the specific cytoskeletal and cell-matrix components were different, including talin 1, collagen type XII α-1 chain, emilin-1, prolyl 4-hydroxylase subunit β (Table 2). These proteins all have integrin binding activities, according to our bioinformatic interaction analysis using the g: Profiler tool. Of the nine proteins that increased, six (RPS9, NOP2, MRTO4, UTP6, PWP2, KRR1) are involved in the metabolism and processing of rRNA, and represent potential novel vimentin-associated proteins. Tubacin treatment reverted the levels of EEF1B2 in the metastasising cells to the levels of the control cells. In contrast, in the metastasising cells, tubacin enhanced the increase in GTP-binding protein 4 (GTPBP4), and further reduced the reduced levels of collagen α-1 (XII) chain, heparan sulphate proteoglycan 2/perlecan, dynamin-like GTPase 1 (MX1), the anion-selective ion channel proteins VDAC1 and VDAC2, and the HLA class I histocompatibility antigens (HLA-A, HLA-B) (Table 2, Figure 4C). The relative levels of vimentin were unchanged across samples (Figure S7). 

## 3. Discussion

The observation that during the metastatic transformation of cells the spatial organisation of vimentin intermediate filaments is more pronouncedly changed than that of microfilaments or microtubules highlights the possibility that vimentin has a more important role in the control of the cell–extracellular matrix adhesion and the cell shape and motility of metastasising cells than previously recognised. 

This proteome analysis of the vimentin-enriched intermediate filament fraction of cells provides a global view of the vimentin interactome and indicates protein pathways and networks for future investigations. It is important to note that these interactions need to be validated using separate methods, and that differences in protein levels between interactomes does not necessarily mean that the binding of a protein to vimentin is different, because this might simply be caused by different total levels of the proteins in cells. For example, we observed reduced levels of collagen 1 and lysyl hydroxylase in the vimentin interactome of metastasising cells, as compared to the immortalised control cells; however, this is likely due to reduced expression of these genes in the metastasising cells [25]. In contrast, the reduced levels of thymopoietin might not be due to reduced gene expression, because the expression of this gene is increased in metastasising cells, as compared to controls. Similarly, the gene expression of the protein that showed the greatest increase in the vimentin interactome of metastasising cells was reduced on the gene expression level—keratin 18 [25]. This highlights the possibility that in metastasising fibroblasts, vimentin can co-assemble with keratin 18 to form an intermediate filament variant and a cell phenotype that is intermediate between those of mesenchymal and epithelial cells. This is in line with previous reports that the co-assembly of keratin 18 and vimentin at subcellular regions in epithelial cells during EMT results in increased cell motility [27]. The muscle actin α1 was identified in the vimentin interactomes of all four of our cell variants and their treatments. This supports earlier observations showing that muscle actins can be incorporated into the cytoskeleton of fibroblasts [33]. Similarly, the actin crosslinkers filamin A and B were identified in all vimentin interactomes (Supplementary Dataset S1). Cells that lack filamin A show defective growth of focal adhesions, which is a phenotype that can be reversed to control cell levels by expression of a calpain-uncleavable, but not full-length, variant of talin 1 [14]. Calpain-cleavage of talin 1 has been shown to be a rate-limiting step in the turnover of cell-matrix adhesions [15,34]. These observations are in line with the model that suggests that competition between talin 1 and filamin A for binding to integrins that is controlled by calpain-mediated proteolysis is required for cell-matrix adhesions to grow [15]. We speculate that vimentin has a major role in this control, through the protection of cell-adhesion components against calpain-mediated proteolysis.

Although inhibition of HDAC6 activity in the metastasising cells resulted in a reversal of the changes to cell shape, cell migration speed and directionality, and the speed–directionality coupling of cell migration compared to control cells, this was not accompanied by a similar reversal of the protein interactions of the vimentin interactome in the metastasising cells. This is not surprising, given that tubacin treatment does not affect gene expression [22], and can therefore not be expected to fully reverse all of the oncogene-induced changes of the transcriptome and the proteome to that of the control cells. Because tubacin targets HDAC6 activity, and not protein levels, our study suggests that it is the activity, and not the levels, of HDAC6 that regulates the observed changes. The inhibition of HDAC6 resulted in a loss of binding of extracellular matrix proteins to vimentin. However, given the short timeframe of the tubacin treatment, changes in the deposition and reorganisation of the extracellular matrix are not expected to be the main contributions to the observed effects on cell shape and motility. The specific binding of talin 1 to vimentin was recently reported by Ostrowska-Podhorodecka et al. [12]; they observed that talin 1 colocalised to filamentous vimentin, and not to small fragments of vimentin, and that vimentin suppressed talin 1-dependent activation of integrin-dependent inside-out signalling, to promote outside-in signalling, focal adhesion growth and maturation. The loss of vimentin further reduced the speed of cell migration in a scrape wound assay. This indicates that by binding to and suppressing the talin 1-dependent activation of integrins, vimentin can promote outside-in signalling, growth of cell-matrix adhesions and the speed of cell migration. This concept is in line with our observations that increased levels of talin 1 in the vimentin interactome of metastasising cells are accompanied by increased cell migration speed and speed–directionality coupling, as compared to tubacin-treated cells. In contrast, the metastasising cells showed reduced directionality as compared to tubacin-treated cells. This highlights that the mechanisms by which vimentin regulates speed and directionality are separate and are mediated through different molecular mechanisms. In addition to talin 1, tubacin reduced the levels of the small Rho GTPase Rab5C in the vimentin interactome, as well as Rab GDP dissociation inhibitor β (GDI2), a GDI that inhibits Rab5C. Rab5C is required for focal adhesion formation, as it activates the calcium-dependent protease calpain 2 and increases the turnover of focal adhesion proteins, including talin 1, to promote cell migration [32,35]. It is therefore possible that GDI-inactivated Rab5C is released from vimentin upon tubacin treatment, to result in increased outside-in signalling over focal adhesions. Tubacin also reduced the levels of the cation membrane transport protein ATP1A1 in the vimentin interactome of the metastasising cells. It is therefore possible that tubacin can increase the electrochemical gradient of cations across the plasma membrane, to induce calpain-mediated proteolysis and focal adhesion turnover and loss. 

We have previously shown that in these metastasising cells, long vimentin fibres localise to the base of mature focal adhesions, and shorter vimentin fragments to the vicinity of small focal complexes, which further supports the concept that long vimentin filaments promote focal adhesion maturation and growth [11]. It is important to note that in these metastasising fibroblasts, vimentin does not show increased localisation to cell adhesion components (e.g., integrin and talin 1) within, as compared to between, focal adhesions [36]. Rather, vimentin is enriched in the proximity of, and not inside, cell-matrix adhesions. This indicates that the role of the binding of cell-adhesion components to vimentin in this cell system might be to sequester these proteins, to provide protection against proteolysis, and for their storage and timely release to control and fine-tune their functions during focal-adhesion formation and turnover, for cell shape and migration. This is in agreement with the previous observation that with treatment with Phorbol 12, 13-dibutyrate, focal adhesions rearrange into podosomes, as a distinct form of cell-matrix adhesions that first appear in subcellular areas with mature and long vimentin filaments [37].

The observation that the inhibition of HDAC6 activity results in nanoscale vimentin filaments at the very periphery of cells, and vimentin clusters and knots that are similar to small, peripheral aggresomes, is in line with previous observations that HDAC6 activity is required for the formation of perinuclear aggresomes in cells. It is therefore likely that HDAC6 activity is required for the retrograde transport of vimentin towards the nucleus that precedes the formation of the perinuclear aggresome, but not for the formation of vimentin knots or asters around defective proteins per se. This is consistent with the recent observation that vimentin is required for the localisation of proteasomes to perinuclear aggresomes [38]. We would like to highlight that the focus on HDAC6 in this study was not dictated by evidence that other HDACs do not regulate vimentin, cell adhesion or migration, but simply because of the cytoplasmic localisation of HDAC6. We have previously shown that the speed of cell migration is positively correlated with the directionality of cell migration in a wide variety of cell types [21,39]. In contrast, these fibroblasts showed a negative correlation between speed and directionality—a correlation which was significantly increased in the oncogene-expressing and metastasising cells, compared to the control cells, and reversed to that of control cells upon inhibition of HDAC6. This suggests that during the transformation of control cells to metastasising cells, a HDAC6-mediated control that ensures high speed only in directional cells is lost. We have previously shown that these metastasising cells show smaller cell-matrix adhesions and a more homogenous distribution of nanoscale adhesion complexes compared to control cells, and that these changes are accompanied by more a entangled nanoscale distribution of the vimentin filaments [18]. Therefore, we speculate that in these metastasising cells, binding to vimentin of components of cell-matrix adhesions induces calpain-mediated focal-adhesion turnover, with a shift from outside-in towards inside-out focal-adhesion signalling. This can suppress the maturation of stable and large focal adhesions that is required for elongated cell shape and directional migration, and instead results in a HDAC6-dependent, dysregulated and non-directed type of migration, which promotes metastasis. 

In summary, we have identified a HDAC6-dependent control of increased speed, reduced directionality, and increased speed–directionality coupling of the migration of metastasising cells. Furthermore, we have suggested the molecular mechanisms of this control, by linking it to the vimentin interactome and the spatial distribution of vimentin, while also indicating novel vimentin-binding proteins. These findings are in line with the hypothesis that the binding of cell-matrix components to vimentin governs their functions, and they offer new avenues for investigations of how vimentin regulates cell–extracellular matrix adhesion, motility and metastasis.

## 4. Materials and Methods

### 4.1. Cell Clture and Treatments

The transformed and metastasising fibroblasts used here were previously constructed by Hahn et al. by the insertion of three well-defined genetic elements: the telomerase catalytic subunit (hTERT) in combination with two oncogenes (the simian virus 40 large-T oncoprotein; an oncogenic allele of H-ras, V12) into neonatal Bj human dermal fibroblasts [23]. These provided the control Bj primary cells, immortalised Bjhtert cells, cell cycle-defective BjhtertSV40T cells, and the final BjhtertSV40TRasV12 variant that forms tumours that metastasise to lungs in immunodeficient mice. These cell variants can be considered as an isogenically matched model system of the transformation of primary cells to metastasising cells, and they have been characterized previously with regard to their total gene transcription and protein expression, as well as the cytoskeletal organisation and behaviour of the cells [18,25,40]. The cells were cultured as described previously [40]. BjhTertSV40TRasV12 fibroblasts were treated with 10 μM tubacin (SML0065; SigmaAldrich, St. Louis, MO, United States) or dimethylsulphoxide and incubated at 37 °C for 4 h.

### 4.2. Immunofluorescence Staining, Confocal and STED Imaging and Computational Analysis of STED Images

The cells were immunostained and analysed under a microscope (AxioVert 40 CFL; Zeiss, Oberkochen, Germany). For dual colour stimulated emission depletion (STED) microscopy, the cells were imaged using a custom-built high-resolution STED microscope with a resolution of 40 nm, as described previously [3]. For triple-colour STED, the cells were transfected with an EGFP variant fused to wild-type vimentin and imaged as described previously [11].

### 4.3. Live-Cell Imaging 

The cells were seeded at 1500 cells/well in slide chambers (543079; Greiner Bio-One Frickenhausen, Germany) and left to grow for 4 days in Dulbecco’s modified Eagle’s medium supplemented with 10% foetal bovine serum and 100 U/mL penicillin and 100 microg/mL streptomycin. Just prior to live-cell imaging, the medium was replaced with fresh medium containing 0.05 μg/mL Hoechst (H3570; Invitrogen, Waltham, MA, United States) with 10 μM tubacin (SML0065; Sigma Aldrich, St. Louis, MO, United States) or dimethylsulphoxide (BP231-100; Thermo Fisher Scientific, Waltham, MA, United States). This was followed by 24 h live-cell imaging under a microscope (Cell Discoverer 7; Zeiss, Oberkochen, Germany), with images taken every 45 min.

### 4.4. Invasion Assay

Matrigel in vitro cell invasion assays were performed in duplicate. A total of 5000 cells were seeded/well in a BD Biocoat Matrigel Invasion 24-well plate chambers (BD Biosciences, Franklin Lakes, NJ, United States) and incubated for 22 h. The number of invading cells per 10× objective field of view were counted according to the manufacturer’s protocol. In parallel, the cells were seeded on tissue-culture plastic at 30 cells per mm^2^. Then, 22 h later, the cells were trypsinized and counted using a Beckman Coulter cell counter and their proliferation rates were determined. The differences in invasion between the cell lines were normalized to this difference in proliferation rates.

### 4.5. Image Analysis

Cell shape was quantified using FIJI version 1.53 (https://imagej.net/software/fiji/ (accessed on 12 October 2021)). For immunofluorescence, the images were thresholded using phalloidin staining; individual cells were selected by using the wand tracing tool and measured for shape descriptors. For quantification from images captured during live-cell imaging, images taken after 4 h were calibrated using a scale bar, and cell shape and area were quantified by manually drawing around individual cells with the free-hand selection tool. 

### 4.6. Statistical Analysis

The statistical analysis of aspect ratios was performed in GraphPad Prism version 9.1.0 (GraphPad Prism Software, San Diego, CA, United States). All of the data were non-parametric, and therefore Mann–Whitney U tests were used to compare the experimental groups. For the migration analysis, Student’s t-tests were used to determine the *p*-values between experimental conditions. A python script was written to calculate the *p*-values using the scipy.stats module and its function ttest_ind() (documentation for ttest_ind(): https://docs.scipy.org/doc/scipy/reference/generated/scipy.stats.ttest_ind.html (accessed on 15 October 2021)). The correlation between the mean migratory speed and the persistence of each cell and under condition was obtained using pythons scipy.stats module using the pearsonr() function (documentation for pearsonr(): https://docs.scipy.org/doc/scipy/reference/generated/scipy.stats.pearsonr.html (accessed on 15 October 2021).

### 4.7. Preparation of Enriched Intermediate Filament Protein Fraction

Cytoskeletal isolation was performed using the method of Herrmann et al., with the modification of using a mass spectrometry-compatible MS-SAFE Protease and Phosphatase Inhibitor Cocktail (Sigma-Aldrich, St. Louis, MO, United States) [41,42,43]. This yielded four subcellular fractions: membrane bound/soluble protein (fraction 1); cytoskeletal (fraction 2); high salt-soluble supernatant (fraction 3) and the insoluble cytoskeleton (fraction 4). Fraction 4 was dissolved in 10 M urea. Although fractions 2 and 4 contained the insoluble proteins, fraction 4 was the fraction of choice for the analysis of intermediate filament proteins, as the levels of other non-specific species were significantly reduced [42,43].

### 4.8. Western Blotting

The cytoskeletal isolation fractions were separated using 4% to 15% precast polyacrylamide gels (4568084; Bio-Rad, Hercules, CA, United States). The gels were then transferred onto nitrocellulose membranes (1704271; Bio-Rad, Hercules, CA, United States) using a transfer system (Trans-Blot Turbo; 1705150; Bio-Rad, Hercules, CA, United States). The blocking and developing of the membranes were carried out as previously described [44]. The antibodies used were as follows: anti-vimentin (V6389; Sigma Aldrich, St. Louis, MO, United States), pan-cytokeratin (ab8068; Abcam, Cambridge, UK), anti-β-actin (A1978; Sigma Aldrich, St. Louis, MO, United States) and Horseradish-peroxide-conjugated goat anti-mouse secondary antibodies (GtxMu-004-DHRPX; ImmunoReagents, Raleigh, NC, United States). Membranes were stripped after imaging vimentin and pan-keratin, and exposed for 2 min to confirm that the membranes had been stripped, before incubating with β-actin antibody as a loading control.

### 4.9. Quantitative Proteomic, Mass Spectrometry-Based Analysis of the Vimentin Interactome

Five micrograms of each fraction 4 sample, with two biological replicates of each, were diluted to 1 M urea using 50 mM NH_4_HCO_3_. The samples were reduced and alkylated by the sequential addition of 5 mM dithiothreitol and 15 mM iodoacetamide, for 30 min at room temperature and 20 min on ice, respectively. Proteins were proteolytically digested using trypsin at a ratio of 1:50, overnight at 37 °C. Following desalting using Hypercarb and C18 tips, peptides were analysed by nano-LC–MS/MS. A chromatography system was used (RSLCnano; Thermo Fisher Scientific, Waltham, MA, United States) with two solvents: solvent A (0.1% formic acid in water) and solvent B (0.08% formic acid in 80% acetonitrile). Peptides were dissolved in 0.1% (*v*/*v*) trifluoroacetic acid and 3% (*v*/*v*) acetonitrile, and two biological replicates were analysed in triplicate in randomised orders. Peptides were resolved on an C18 column (EASY-Spray PepMap RSLC; 50 cm × 75 μm ID, 2 μm; 40 °C; Thermo Fisher Scientific, Waltham, MA, United States) with a gradient programme delivered at 300 nL/min with 0–5 min at 3% B, then increasing from 3% B to 50% B over the next 30 min. Peptides were analysed by a mass spectrometer (Q Exactive HF hybrid quadrupole-Orbitrap; Thermo Fisher Scientific, Waltham, MA, United States) that was programmed to be data-dependent with 10 product ion scans (centroid: resolution, 30,000; automatic gain control, 1 × 10^5^ maximum injection time, 60 ms; isolation: normalized collision energy, 27; intensity threshold, 1 × 10^5^) per full mass spectrometry scan (profile: resolution, 120,000; automatic gain control, 1 × 10^6^; maximum injection time, 60 ms; scan range, 375–1500 *m/z*). 

### 4.10. Protein Identification, Relative Quantification, Bioinformatic Functional Profiling and Interaction Analysis

Proteins were identified by searching the mass spectrometry data files against the *Homo sapiens* proteome database (www.uniprot.org/proteomes/UP000005640, downloaded, 2 August 2021; 78120 entries) using MaxQuant v. 1.6.4.0 with the label free quantification (LFQ) and intensity-based absolute quantification options selected [45,46,47]. Default settings were used with search parameters set to include the following modifications: carbamidomethyl-Cys (fixed); Met oxidation; protein N-terminal acetylation (variable); maximum of two missed tryptic cleavages. Peptide-spectrum matches and protein identifications were filtered using a target-decoy approach at a false discovery rate (FDR) of 1%. Statistical analyses were performed using LFQAnalyst (bioinformatics.erc.monash.edu/apps/LFQ-Analyst/, accessed 18 October 2021), where the LFQ intensity values were used for protein quantification [48]. Missing values were replaced by values drawn from a normal distribution of 1.8 standard deviations and a width of 0.3 for each sample (Perseus-type). Protein-wise linear models combined with empirical Bayesian statistics were used for differential expression analysis using the Bioconductor package limma, whereby the adjusted P-value cut-off was set at 0.05 and log2 fold change cut-off was set at 1. The Benjamini–Hochberg method of FDR correction was applied. The g: Profiler tool was used for functional enrichment analysis g:Profiler (version e104_eg51_p15_3922dba) with the Benjamini-Hochberg FDR method applying a significance threshold of 0.05 [44]. The Min and Max size settings of the functional category were set to 3 and 500, respectively, with No electronic gene ontology (GO) annotations selected. GO terms for Molecular Function, Biological Process, Cellular Compartment, Kyoto Encyclopedia of Genes and Genomes (KEGG) and Reactome were assigned. The species was set to *Homo sapiens*. 

### 4.11. Tracking and Analysis of Migrating Cells

Cell migration was analysed by tracking the cell nuclei using the Fiji plug-in of TrackMate [49]. The full documentation of this plug-in can be seen on the ImageJ wiki: https://imagej.net/plugins/trackmate/ (accessed on 15 October 2021). The following specific settings were chosen for the tracking: Detector, Difference of Gaussian (Dog); estimated object size, 20 µm; quality threshold, 2.0; duration of tracks, min. 18 h. All other settings remained at their default options. The tracks acquired were analysed using the in-built algorithms (https://imagej.net/plugins/trackmate/algorithms, accessed on 15 October 2021). 

## Figures and Tables

**Table 1:** Changes in protein levels for all of the proteins altered in the vimentin interactome of metastasising BjhtertSV40TRasV12, relative to immortalised Bjhtert cells.

Protein	Gene	Protein ID	BjhtertSV40TRasV12 vs. Bjhtert (Fold-Change) ^a^
Protein SON	*SON*	P18583	5.5
Neuroblast differentiation-associated protein AHNAK	*AHNAK*	Q09666	4.7
WD repeat-containing protein 46	*WDR46*	O15213	4.5
60S ribosomal protein L7-like 1	*RPL7L1*	Q6DKI1	4.2
Ribosome biogenesis protein BOP1	*BOP1*	Q14137	4.1
RNA-binding protein 8A	*RBM8A*	Q9Y5S9	4.0
Transcription factor BTF3	*BTF3*	A0A7I2YQL2	3.7
Triosephosphate isomerase	*TPI1*	P60174	3.7
Elongation factor 1-β	*EEF1B2*	P24534	3.6
Keratin, type I cytoskeletal 18	*KRT18*	P05783	3.5
U3 small nucleolar RNA-associated protein 15 homologue	*UTP15*	Q8TED0	3.2
WD repeat-containing protein 75	*WDR75*	Q8IWA0	3.2
Guanine nucleotide-binding protein-like 3	*GNL3*	Q9BVP2	3.1
Nuclear pore complex protein Nup153	*NUP153*	P49790	3.1
DNA-directed RNA polymerase II subunit RPB3	*POLR2C*	P19387	3.0
U3 small nucleolar ribonucleoprotein protein MPP10	*MPHOSPH10*	O00566	2.9
ATP-dependent RNA helicase DDX18	*DDX18*	Q9NVP1	2.9
Deoxynucleotidyltransferase terminal-interacting protein 2	*DNTTIP2*	Q5QJE6	2.8
Ribosome production factor 2 homologue	*RPF2*	Q9H7B2	2.6
Ras-related protein Rab5C	*RAB5C*	P51148	2.6
H/ACA ribonucleoprotein complex subunit 4	*DKC1*	O60832	2.5
MKI67 FHA-domain-interacting nucleolar phosphoprotein	*NIFK*	Q9BYG3	2.4
Ribosome biogenesis protein BMS1 homologue	*BMS1*	Q14692	2.2
Nucleolar GTP-binding protein 1	*GTPBP4*	Q9BZE4	2.2
Voltage-dependent anion-selective channel protein 2	*VDAC2*	P45880	−2.1
Histone H2B type 2-E	*HIST2H2BE*	Q16778	−2.1
RuvB-like 1	*RUVBL1*	Q9Y265	−2.2
Actin, α skeletal muscle	*ACTA1*	P68133	−2.2
Bcl-2-associated transcription factor 1	*BCLAF1*	A0A1W2PQ43	−2.2
Testis-expressed sequence 10 protein	*TEX10*	Q9NXF1	−2.5
HLA class I histocompatibility antigen, Cw-7 α chain	*HLA-C.1*	O19617	−2.5
CD109 antigen	*CD109*	Q6YHK3	−2.6
Collagen α-1(XII) chain	*COL12A1*	D6RGG3	−2.6
HLA class I histocompatibility antigen, A-3 α chain	*HLA-A*	Q5SUL5	−2.6
Procollagen-lysine,2-oxoglutarate 5-dioxygenase 1/lysyl hydroxylase	*PLOD1*	Q02809	−2.6
Heterogeneous nuclear ribonucleoprotein H3	*HNRNPH3*	P31942	−2.8
Lamina-associated polypeptide 2, isoform α/ thymopoietin	*TMPO*	P42166	−2.9
WD repeat-containing protein 18	*WDR18*	U3KQC1	−2.9
Heterogeneous nuclear ribonucleoprotein U-like protein 2	*HNRNPUL2-BSCL2*	H3BQZ7	−3.1
HLA class I histocompatibility antigen B α chain	*HLA-B*	Q2L6G2	−3.2
Voltage-dependent anion-selective channel protein 1	*VDAC1*	P21796	−3.2
Fibulin-2	*FBLN2*	P98095	−3.5
Basement membrane-specific heparan sulphate proteoglycan core protein	*HSPG2*	P98160	−4.1
Interferon-induced GTP-binding protein Mx1	*MX1*	A0A7P0T9R0	−4.4
26S proteasome non-ATPase regulatory subunit 6	*PSMD6*	Q15008	−4.6
Myosin regulatory light chain 12A	*MYL12A*	J3QRS3	−6.8
Interferon-induced GTP-binding protein Mx2	*MX2*	P20592	−8.9

^a^ positive value, increase; negative value, decrease.

**Table 2:** Tubacin effects (i.e., inhibition of HDAC6 activity) for all of the proteins altered in the vimentin interactome of metastasising BjhtertSV40TRasV12 cells.

Protein	Gene	Protein ID	BjhtertSv40TRasV12 with vs. without Tubacin (Fold-Change) ^a^
40S ribosomal protein S23	*RPS23*	P62266	2.1
GTP-binding protein 4	*GTPBP4*	Q9BZE4	2.1
40S ribosomal protein S9	*RPS9*	P46781	2.2
Probable 28S rRNA (cytosine(4447)-C(5))-methyltransferase	*NOP2*	P46087	2.3
mRNA turnover protein 4 homologue	*MRTO4*	Q9UKD2	2.5
Probable ATP-dependent RNA helicase DDX56	*DDX56*	G3V0G3	2.7
U3 small nucleolar RNA-associated protein 6 homologue	*UTP6*	Q9NYH9	3.1
Periodic tryptophan protein 2 homologue	*PWP2*	Q15269	4.3
KRR1 small subunit processome component homologue	*KRR1*	Q13601	4.9
26S protease regulatory subunit 10B	*PSMC6*	A0A087X2I1	−2.0
Glycine--tRNA ligase	*GARS*	A0A6Q8PGW4	−2.1
Transitional endoplasmic reticulum ATPase	*VCP*	P55072	−2.1
X-ray repair cross-complementing protein 6	*XRCC6*	P12956	−2.1
Eukaryotic translation initiation factor 5B	*EIF5B*	A0A087WUT6	−2.1
T-complex protein 1 subunit γ	*CCT3*	B4DUR8	−2.1
Emilin-1	*EMILIN1*	Q9Y6C2	−2.3
Glucose-6-phosphate 1-dehydrogenase	*G6PD*	P11413	−2.4
Prohibitin	*PHB*	P35232	−2.5
Rab GDP dissociation inhibitor β	*GDI2*	P50395	−2.5
Tubulin-specific chaperone A	*TBCA*	E5RIW3	−2.5
Dolichyl-diphospho-oligosaccharide--protein glycosyltransferase 48 kDa subunit	*DDOST*	A0A0C4DGS1	−2.6
26S proteasome non-ATPase regulatory subunit 2	*PSMD2*	Q13200	−2.6
Malate dehydrogenase, mitochondrial	*MDH2*	P40926	−2.7
mRNA export factor	*RAE1*	E9PQ57	−2.7
Coatomer subunit γ-1	*COPG1*	Q9Y678	−2.8
Voltage-dependent anion-selective channel protein 2/voltage dependent anion channel 2	*VDAC2*	P45880	−2.9
Heterogeneous nuclear ribonucleoprotein R	*HNRNPR*	O43390	−2.9
Heat shock protein β-1	*HSPB1*	P04792	−3.0
Elongation factor 1-β	*EEF1B2*	P24534	−3.1
Neutral α-glucosidase AB	*GANAB*	Q14697	−3.1
Talin-1	*TLN1*	Q9Y490	−3.1
Peroxiredoxin-6	*PRDX6*	P30041	−3.2
Sodium/potassium-transporting ATPase subunit α-1	*ATP1A1*	P05023	−3.2
HLA class I histocompatibility antigen B α chain	*HLA-B*	Q2L6G2	−3.3
Flotillin-1	*FLOT1*	O75955	−3.4
Leucine-rich repeat-containing protein 59	*LRRC59*	Q96AG4	−3.4
tRNA-splicing ligase RtcB homologue	*RTCB*	Q9Y3I0	−3.4
Transcription intermediary factor 1-β	*TRIM28*	Q13263	−3.5
Serpin H1	*SERPINH1*	P50454	−3.6
Interferon-induced GTP-binding protein Mx1/dynamin like GTPase 1	*MX1*	A0A7P0T9R0	−3.6
Signal transducer and activator of transcription 1-α/β	*STAT1*	A0A669KB68	−3.8
Protein CutA	*CUTA*	O60888	−3.9
Protein disulphide-isomerase/collagen prolyl 4-hydroxylase β	*P4HB*	A0A7P0T8J3	−4.3
Basement-membrane-specific heparan sulphate proteoglycan core protein	*HSPG2*	P98160	−4.6
KH domain-containing, RNA-binding, signal transduction-associated protein 1	*KHDRBS1*	Q07666	−4.6
Collagen α-1(XII) chain	*COL12A1*	D6RGG3	−4.8
Tropomyosin α-4 chain	*TPM4*	P67936	−4.8
Voltage-dependent anion-selective channel protein 1	*VDAC1*	P21796	−4.9
Prothymosin α	*PTMA*	B8ZZQ6	−5.0
Protein S100-A11	*S100A11*	P31949	−5.4
C-1-tetrahydrofolate synthase, cytoplasmic	*MTHFD1*	V9GYY3	−5.7
HLA class I histocompatibility antigen, A-3 α chain	*HLA-A*	Q5SUL5	−11.6
RPLP1	*RPLP1*	P05386	−50.6

^a^ positive value, increase; negative value, decrease.

## Data Availability

The data presented in this study are available in the main text, references, and Supplementary Information of this Manuscript, and in the Human Protein Atlas (https://www.proteinatlas.org (accessed on 18 October 2021)).

## Supplementary Materials

The following supporting information can be downloaded at: https://www.mdpi.com/article/10.3390/ijms23041961/s1.
