# Supplementary material for: Metastasising Fibroblasts Show an HDAC6-Dependent Increase in Migration Speed and Loss of Directionality Linked to Major Changes in the Vimentin Interactome
Source: Int J Mol Sci. 2022 Feb 10;23(4):1961. doi: 10.3390/ijms23041961 (PMC8880509; doi:10.3390/ijms23041961)
Supplement: Supplementary file 1 [file ijms-23-01961-s001.zip › Supplementary Files.pdf]

Supplementary figures

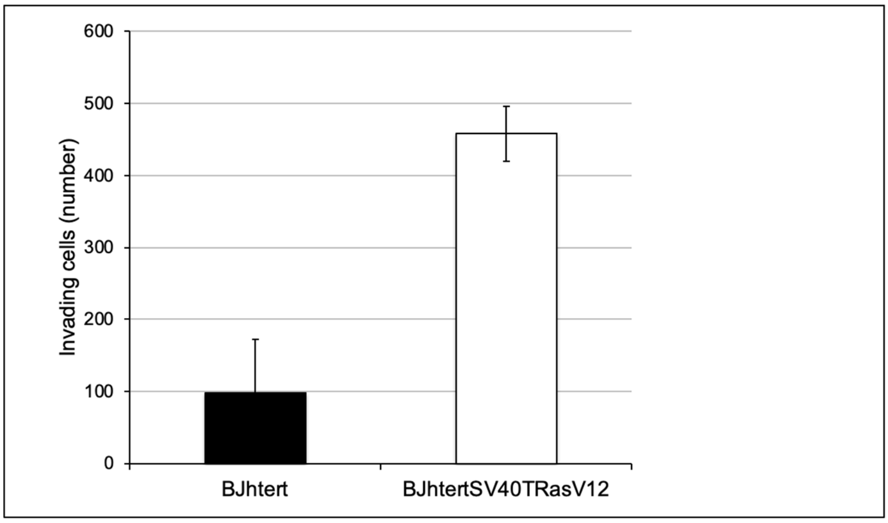

Figure S1. Invasion into the Extracellular matrix of Bj fibroblast variants. Graph showing the numbers of invading Bjhtert and BjhtertSV40TRasV12 cells per area of vision in a Matrigel *In vitro* cell invasion assay, normalised to proliferation differences, as described in the Material and Method section. Average values of three independent experiments are shown.

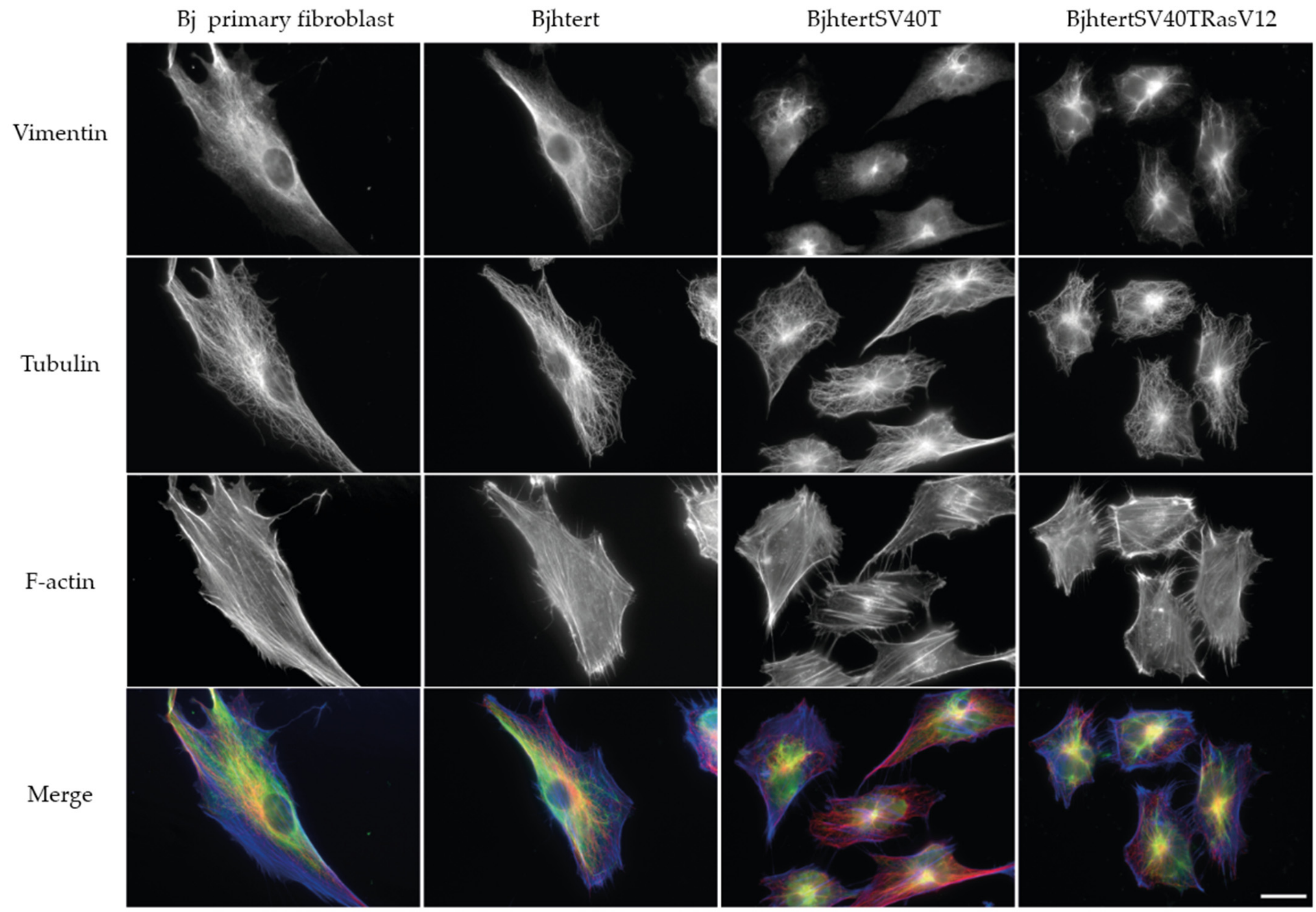

Figure S2. Cytoskeletal organisation of fibroblasts for the different stages of metastatic transformation. Immunoflourescence staining followed by Epiflourescence microscopy showing Bj primary fibroblasts, Bjhtert, BjhtertSV40T, BjhtertSV40TRasV12 cells, with vimentin, F-actin and tubulin as single colours, as indicated, and merged colour images showing vimentin (green), tubulin (red) and F-actin (blue). Scale bar: 10 µm.

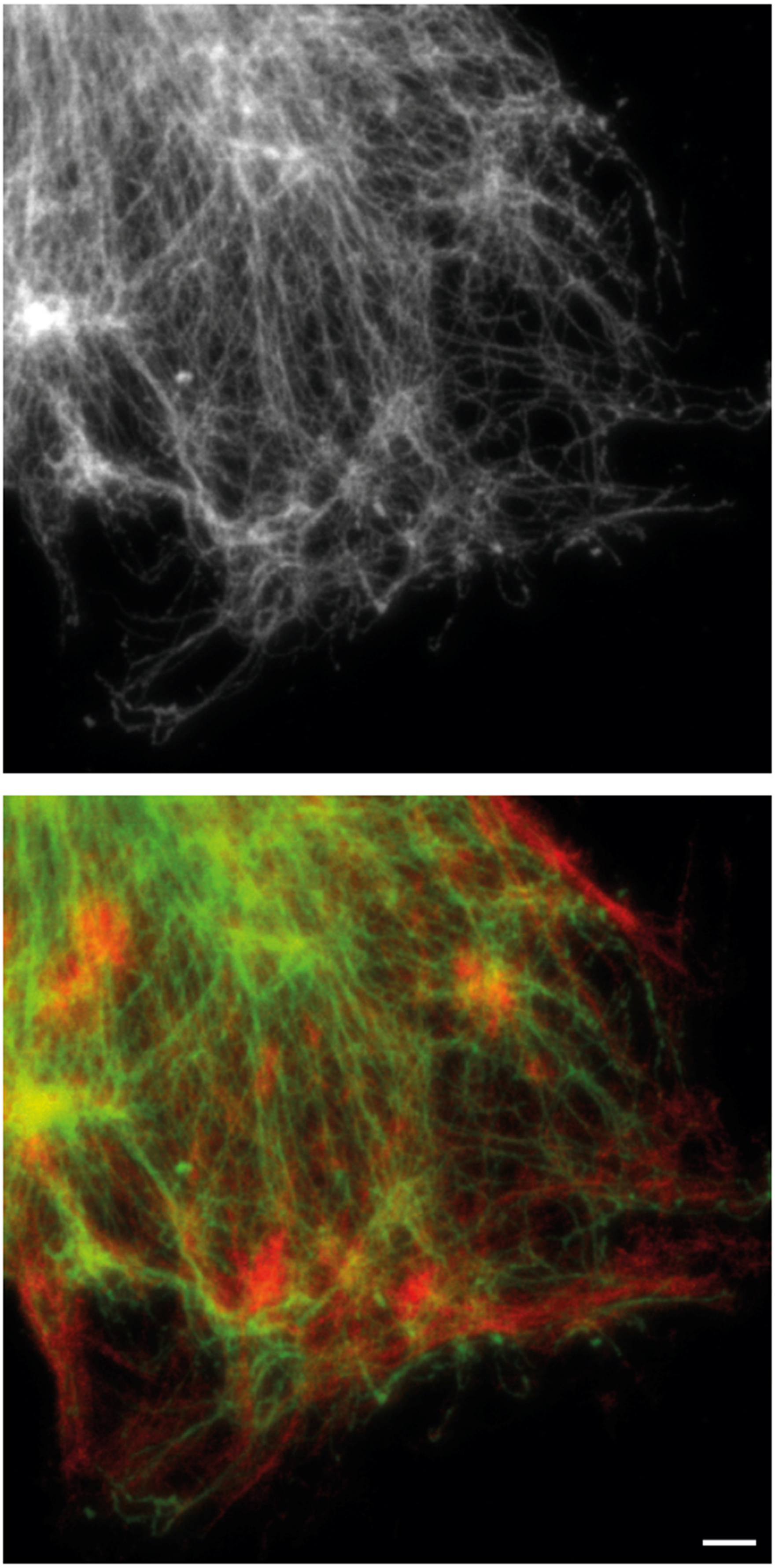

Figure S3: HDAC6 regulates the nanoscale spatial distribution of vimentin in oncogene-expressing and metastasising cells. Representative stimulated emission depletion (STED) super-resolution microscopy images of BjhtertSV40T cells treated with tubacin and immunostained, showing vimentin (top), and vimentin (green) and F-actin (red) (bottom). Scale bar: 1 μm.

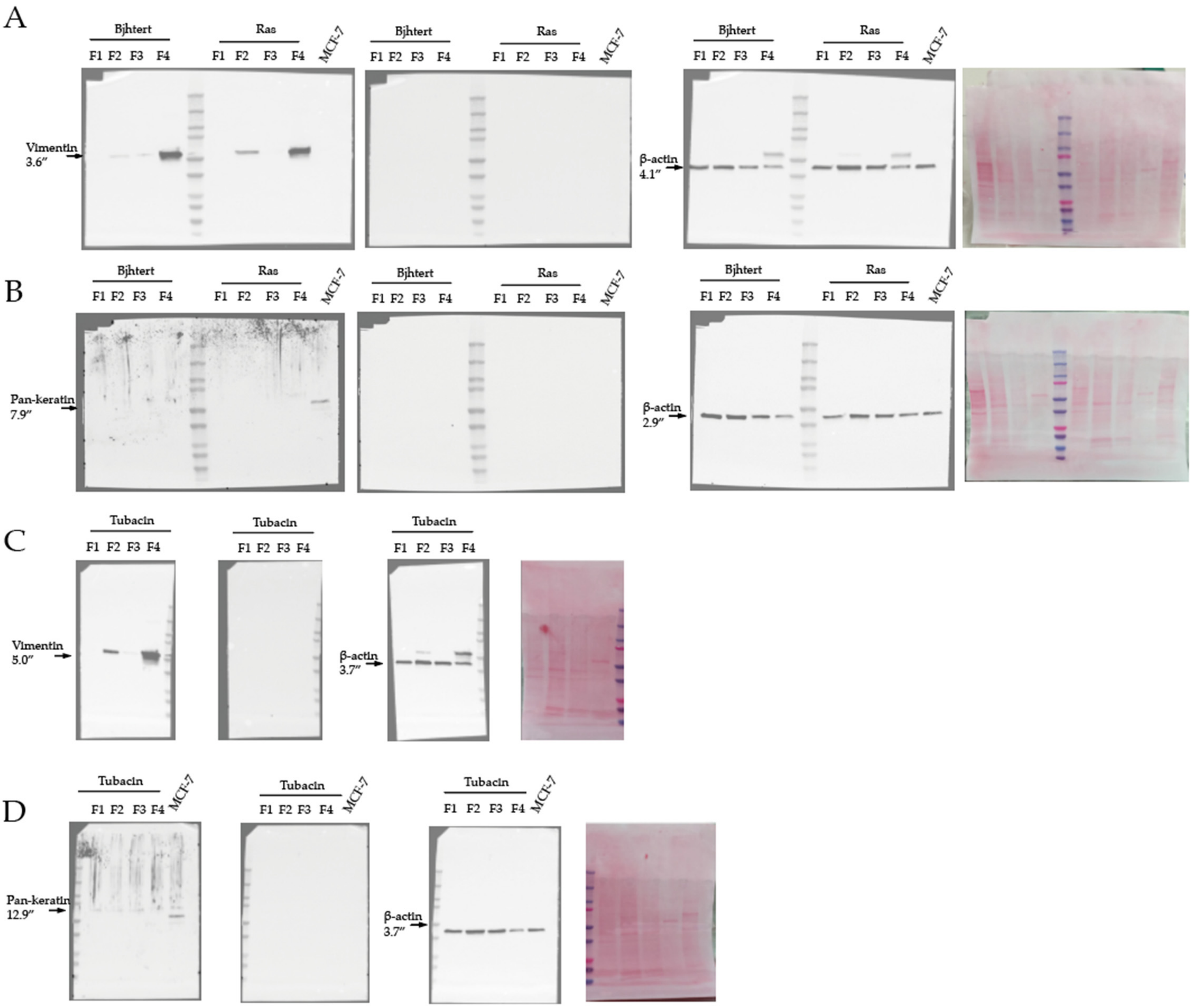

Figure S4. Representatives complete Western blotting membranes and Ponceau-S stained membranes from the four cell fractions. Blot membranes and Ponceau-S staining (far right) shown for Bjhtert cells (A, B) and BjhtertSV40TRasV12 (Ras; A-D) cells without (A, B) and with (C, D) tubacin treatment. (B, D) MCF-7 lysate included as positive control for keratin. C. (C, D) Blots were cut vertically along the centre of the ladder and incubated separately with vimentin (C) and pan-keratin (D) antibodies, respectively. Exposure times are given in seconds (″).

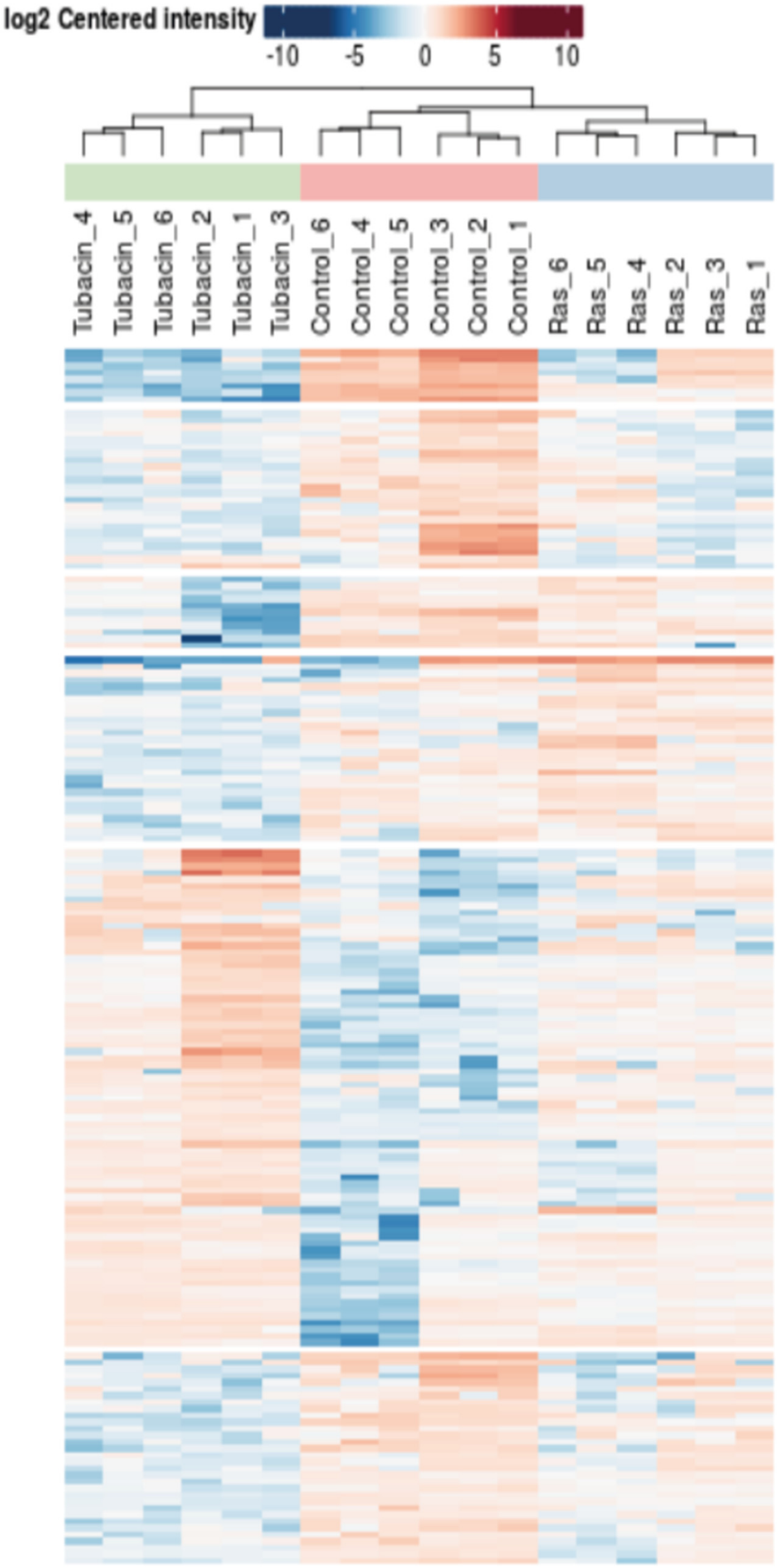

Figure S5: Hierarchical clustering heatmap and principal component analysis of the differentially affected proteins. Mass spectrometry showing Log2 intensity of protein in Bjhtert cells (Control, red) and BjhtertSV40TRasV12 without (Ras, blue) and with (Tubacin, green) tubacin treatment.

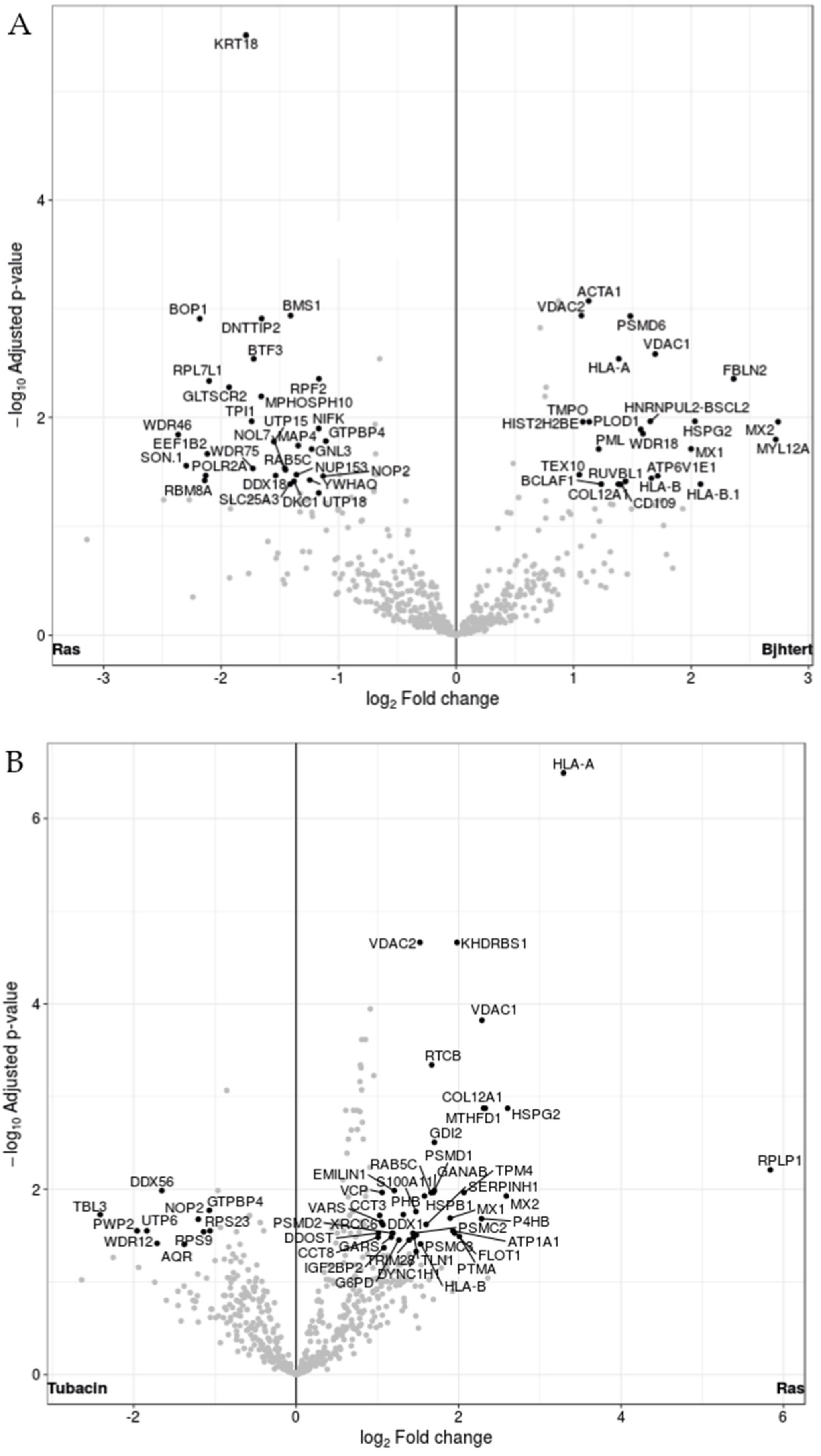

Figure S6: Volcano plots for statistical significance *versus* fold-changes in protein levels. Mass spectrometry showing –Log10 adjusted p-values *versus* Log2 protein fold-changes between Bjhtert cells and BjhtertSV40TRasV12 cells without (Ras) and with (Tubacin) tubacin treatment. (A) Bjhtert *versus* BjhtertSV40TRasV12 cells. (B)

BjhtertSV40TRasV12 cells without versus with tubacin. Statistically significant protein changes (black dots) are indicated (false discovery rate, <0.05).

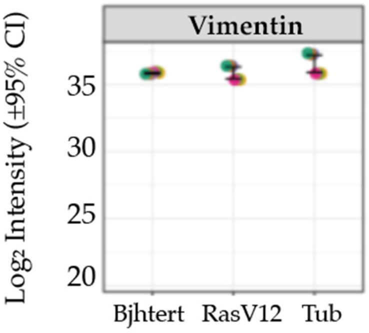

Figure S7: Vimentin protein levels during metastatic transformation and upon inhibition of HDAC6 activity. Mass spectrometry showing protein levels of vimentin in immortalised Bjhtert control cells and metastasising BjhtertSV40TRasV12 cells without (RasV12) and with (Tub) tubacin treatment. The six replicate measurements (two biological replicates, analysed in triplicate) are indicated by the coloured dots for each sample.

Supplementary Table S1: RNA-metabolism-related protein levels that were changed for the metastasising BjhtertSV40TRasV12 cells relative to the control Bjhtert cells.

**Protein**	**Gene**	**Uniprot accession number**	**BjhtertSv40T RasV12 *vs* Bjhtert (fold-change)[a]**
WD repeat-containing protein 46	*WDR46*	O15213	4.5
Ribosome biogenesis protein BOP1	*BOP1*	Q14137	4.1
U3 small nucleolar RNA-associated protein 15 homologue	*UTP15*	Q8TED0	3.2
WD repeat-containing protein 75	*WDR75*	Q8IWA0	3.2
Guanine nucleotide-binding protein-like 3	*GNL3*	Q9BVP2	3.1
U3 small nucleolar ribonucleoprotein protein MPP10	*MPHOSPH10*	O00566	2.9
H/ACA ribonucleoprotein complex subunit 4	*DKC1*	O60832	2.5
Ribosome biogenesis protein BMS1 homologue	*BMS1*	Q14692	2.2
RNA-binding protein 8A	*RBM8A*	Q9Y5S9	4.0
Nuclear pore complex protein Nup153	*NUP153*	P49790	3.1

[a], positive value, increase; negative value, decrease
